# *Lactiplantibacillus plantarum* LPPerfectus001 Alleviating Irritable Bowel Syndrome in Mice by Modulating Gut Microbiota and NF-κB Signaling Pathway

**DOI:** 10.3390/foods15030417

**Published:** 2026-01-23

**Authors:** Yue Wang, Xiaoyue Bai, Yizhi Jing, Xin Feng, Yajuan Guo, Yanling Hao, Dun Su, Zhengyuan Zhai

**Affiliations:** 1 College of Food Science and Nutritional Engineering, China Agricultural University, Beijing 100083, China; wyuee0129@163.com (Y.W.); baixiaoyue21@163.com (X.B.); 15961877791@163.com (X.F.); 2 Key Laboratory of Precision Nutrition and Food Quality, Department of Nutrition and Health, China Agricultural University, Beijing 100193, China; j15502414985@163.com (Y.J.); haoly@cau.edu.cn (Y.H.); 3 Perfect (Guangdong) Co., Ltd., Zhongshan 528451, China; guoyajuan@perfect99.com; 4 Perfect Life and Health Institute Co., Ltd., Zhongshan 528451, China

**Keywords:** irritable bowel syndrome, *Lactiplantibcillus plantarum*, gut microbiota, NF-κB, butyrate

## Abstract

Irritable bowel syndrome (IBS) is a prevalent gastrointestinal disorder, often accompanied by low-grade inflammation, visceral hypersensitivity and gut microbiota dysbiosis. In this study, the therapeutic potential of *Lactiplantibacillus plantarum* LPPerfectus001 (*L. plantarum* 001) was investigated to alleviate IBS symptoms. Using an Lipopolysaccharides (LPS)-induced RAW264.7 macrophage model, *L. plantarum* 001 demonstrated significant anti-inflammatory properties by inhibiting Nitric Oxide production and downregulating pro-inflammatory cytokines. Furthermore, in a mouse model of IBS induced by *Citrobacter rodentium* infection and water avoidance stress, *L. plantarum* 001 intervention reduced fecal moisture, improved intestinal barrier integrity via up-regulating of ZO-1 and MUC2, and attenuated visceral hypersensitivity. Transcriptomic analysis combining with RT-qPCR revealed that *L. plantarum* 001 modulated the NF-κB signaling pathway and Th1/Th2 cell differentiation, reducing expression of key inflammatory genes. Additionally, 16S rRNA sequencing showed that *L. plantarum* 001 restored gut microbiota diversity, enriched beneficial butyrate-producing *Odoribacter*, and suppressed pro-inflammatory *Pseudomonadota*. These findings suggested that *L. plantarum* 001 alleviates IBS through multi-targeted mechanisms involving barrier repair, microbiota modulation, and anti-inflammatory signaling, highlighting its potential as a probiotic therapy for IBS.

## 1. Introduction

Irritable bowel syndrome (IBS) is a common functional gastrointestinal disorder (FGID) that manifests as chronic abdominal discomfort, bloating, and changes in stool form or bowel habits. These symptoms are frequently accompanied by visceral hypersensitivity, persistent low-grade inflammation, and disruptions in gut microbial composition [1,2]. According to the Bristol Stool Form Scale, IBS is categorized into constipation-predominant (IBS-C), diarrhea-predominant (IBS-D), mixed bowel habits (IBS-M), and unclassified (IBS-U) subtypes [3]. IBS represents a global health challenge, with estimated prevalence rates of 8.1% in North America and 9.6% in Asia, significantly diminishing patients’ quality of life and increasing healthcare and socioeconomic burdens [4,5,6]. Current pharmacological treatments for IBS often show severe side effects. Therefore, a safe and effective therapeutic strategy is needed to alleviate both the personal and societal burden of IBS.

Altered gut microbial composition is widely recognized as a key contributor to IBS pathology. Patients frequently exhibit reduced populations of beneficial genera such as *Lactobacillus* and *Bifidobacterium*, combined with increased levels of potentially pathogenic bacteria including *Enterobacteriaceae* and *Bacteroides* [7,8]. Notably, compared with fecal microbiota transplant (FMT) from a healthy donor, FMT from an IBS-D patient into germ-free rat results in an increase in intestinal permeability and bacteria translocation into the portal vein. This FMT experiment highlights the direct contribution of gut microbiota imbalance to the development of IBS [9]. Multiple clinical trials have shown that supplementation with probiotics can significantly improve quality of life of IBS patients [10]. For instance, *Lactiplantibacillus plantarum (L. plantarum*) 299 v has been shown to alleviate abdominal pain and bloating largely through modulation of gut microbiota composition [11].

Accumulating evidence from animal research supports the therapeutic potential of probiotics in IBS. For instance, *Bifidobacterium longum* R0175 and *Lactobacillus helveticus* R0052 synergistically reduced stress-related visceral hypersensitivity through regulation of the Hypothalamic–Pituitary–Adrenal (HPA) axis [12]. Comparative genomics analyses indicated that the alleviation of IBS by *Lactobacillus* might be mediated by the synthesis of conjugated linoleic acid [13]. Furthermore, animal studies have underscored that functional properties can vary substantially even among strains of the same species. *L. plantarum* AR495 was shown to alleviate visceral hypersensitivity by suppressing the overactivated mast cell–PAR2–TRPV1 signaling pathway and maintaining intestinal barrier homeostasis [14]. *L. plantarum* D266 could modulate intestinal physiology and enteric neurons in IBS through microbial tryptophan metabolites [15]. *L. plantarum* CCFM8610 exhibited potent anti-inflammatory effects by downregulating pro-inflammatory cytokines and reinforcing epithelial barrier integrity [16]. These findings suggested that the ability of probiotics to alleviate IBS is highly strain specific.

Low-grade colonic inflammation and aberrant immune activation are now recognized as central features of IBS pathophysiology, with hyperactivation of the nuclear factor-κB (NF-κB) signaling pathway driving pro-inflammatory cytokine production, intestinal barrier disruption, and visceral hypersensitivity [17,18]. In the present study, we initially screened *L. plantarum* strains for anti-inflammatory potential using an Lipopolysaccharides (LPS)-activated RAW264.7 macrophage model. Subsequently, we employed physiological assessments along with transcriptomic profiling in a mouse model of stress-induced IBS to elucidate how *L. plantarum* 001 ameliorates IBS-related symptoms. Our findings demonstrate that *L. plantarum* 001 exerts therapeutic effects through coordinated modulation of gut microbiota, immune responses, and NF-κB signaling

## 2. Materials and Methods

### 2.1. Bacterial Strains and Growth Conditions

In this study, 6 strains of *L. plantarum* isolated from different traditional fermented food were selected as candidate strains. This selection was made to include isolates originating from distinct ecological niches, allowing comparison among strains with potentially diverse biological characteristics. All strains used are listed in Table A1. These strains were inoculated into 2% (*v*/*v*) de Man, Rogosa, and Sharp (MRS) medium (Beijing Aobox Biotechnology Co., Ltd., Beijing, China) and subcultured three times at 37 °C for 24 h. *Citrobacter rodentium* DBS100 was purchased from Ningbo Mingzhou Biotechnology Co., Ltd., (Ningbo, China) and cultured in Luria-Bertani (LB) at 37 °C for 20 h at 200 rpm.

### 2.2. Anti-Inflammatory Assay with RAW 264.7 Macrophages

This LPS-induced inflammation model for evaluating probiotic immunomodulatory activity has been widely applied in previous studies [19]. RAW 264.7 cells (Meisen Chinese Tissue Culture Collections, Jinhua, China) were maintained in RPMI-1640 medium supplemented with 10% fetal bovine serum (FBS), 100 U/mL penicillin, and 0.1 mg/mL streptomycin (Sigma-Aldrich, St. Louis, MO, USA). Cells were incubated at 37 °C with 5% CO_2_ and seeded into 96-well plates at 5 × 10^5^ cells/mL until reaching > 85% confluence. For the anti-inflammatory assay, the cells were first treated with 1 mL DMEM containing 1 × 10^6^ CFU/mL different *L. plantarum* strains for 2 h at 37 °C. After this pretreatment, 10 μL of a 100 μg/mL LPS (Sigma-Aldrich, Natick, MA, USA) working solution was added, and the cells were incubated for an additional 22 h. After treatment, both the cells and supernatants were collected. The concentration of nitric oxide (NO) in the supernatant was measured using Nitric Oxide (NO) Colorimetric Assay Kit (Elabscience, Wuhan, China).

To determine the expression level of pro- inflammatory genes, total RNA was isolated using AG RNAex Pro reagent and reverse-transcribed into cDNA with the Evo M-MLV RT Mix Kit (Accurate Biotechnology, Changsha, China). Gene expression was quantified by real-time quantitative polymerase chain reaction (RT-qPCR) using SYBR Green Premix Pro Taq Hs qPCR kit on a QuantStudio™5 Real-Time PCR Systems (Thermo Fisher, Wilmington, NC, USA). Primer sequences are listed in Table A2.

### 2.3. Animal Experimental Design

Male specific pathogen-free (SPF) C57BL/6J mice (8 weeks, 18–20 g) were purchased from Beijing Vital River Laboratory Animal Technology Co., Ltd. Animals were housed under controlled conditions (25 ± 2 °C, 50 ± 5% humidity, 12 h light/dark cycle). After one-week acclimatization, mice were randomly divided into three groups (*n* = 6): Control, Model, and LP001. This experiment was conducted as described in previous studies [13].

On the first day, Model and LP001 groups received oral gavage with *Citrobacter rodentium* DBS110 (1 × 10^10^ CFU in 0.2 mL PBS), while control mice received PBS only. From day 2 to day 29, LP001 group received daily gavage of *L. plantarum* 001 (2 × 10^9^ CFU in 0.2 mL PBS), the other groups received PBS. From days 2 to 8, all mice received subcutaneous injections of 0.5 mL lactated Ringer’s (LR) solution to prevent dehydration caused by diarrhea. And then the mice were exposed to water avoidance stress (WAS) only in model and LP001 group from day 18 to day 30. All animal procedures were approved by the Institutional Animal Care and Use Committee of China Agricultural University (Approval No. Aw31604202-4-2).

### 2.4. Measurement of Fecal Moisture

Fecal samples were collected on day 28 and immediately weighed. Samples were dried at 60 °C for 48 h before re-weighing. The ratio of the difference between the two fecal weights and the original fecal weight was recorded as the moisture content of feces (MCF).

### 2.5. Visceral Hypersensitivity Assignment

The visceral sensitivity during colorectal distention (CRD) in mice were evaluated using the Abdominal Withdrawal Reflex Scores (AWRs) [20]. Mice were fasted for 16 h, anesthetized with 4% isoflurane, and intubated with a glycerol-lubricated balloon catheter (6Fr, 2 mm outer diameter) inserted 1 cm into the colorectum. The catheter was secured to the tail of the mouse with tapes, and the mouse was placed in a breathable plastic box. In the plastic box, mice can only walk back and forth and cannot turn around. After the mice have adapted for 30 min, the colon and rectum will gradually dilate by inflating and pressurizing. The pressure gradually increases from 0, 0.1, 0.2 to 0.3 mL, with each pressure expanding for 20 s. After the evaluation is completed, deflate the balloon and withdraw it. AWR scoring criteria are shown in Table A2.

### 2.6. Collection of Feces, Blood and Tissue Samples

Fecal samples were gathered and preserved at −80 °C. Subsequently, the mice were euthanized using isoflurane. Serum was extracted and stored at −80 °C. The lengths of the colon and entire intestine were measured and the ratio of colon to total intestinal length was calculated. A segment of the colon was immersed in paraformaldehyde for preservation, and the rest was frozen at −80 °C.

### 2.7. Colon Tissue Staining and Immunofluorescence

The colonic tissue was fixed in 4% paraformaldehyde for 24 h and subsequently embedded in paraffin. The embedded tissue was then sectioned into 4 μm thin slices and stained with hematoxylin and eosin (H&E).

For Alcian blue-periodic acid-Schiff (AB-PAS) staining, dewaxed and hydrated sections were first incubated with Alcian blue solution, followed by periodic acid treatment and Schiff reagent according to standard histological procedures. For goblet cell quantification, two AB-PAS-stained images were analyzed for each group, and twenty crypts per image were counted.

For Immunofluorescence, after dewaxing and hydration, citric acid antigen repair buffer was used to repair antigen of colonic tissue sections. Sections were incubated with primary antibodies against ZO-1 (1:2000, Abcam, Cambridge, UK) and MUC2 (1:300, Proteintech, Rosemont, IL, USA), followed by fluorophore-conjugated secondary antibodies. Nuclei were counterstained with DAPI, and images were captured using confocal microscopy.

### 2.8. Real-Time Quantitative PCR

Approximately 25 mg of colon tissue was homogenized, and RNA was extracted using AG RNAex Pro reagent (Accurate Biotechnology Co., Ltd., Changsha, China). RNA purity was verified via NanoDrop ONEc Spectrophotometer (Thermo Fisher Scientific, Waltham, MA, USA). 1 µg of RNA was reverse-transcribed using the Evo M-MLV RT Mix Kit (Accurate Biotechnology Co., Ltd., Changsha, China).

RT-qPCR was performed using SYBR Green Premix Pro Taq Hs qPCR kit (Accurate Biotechnology Co., Ltd., Changsha, China) with QuantStudio™5 Real-Time PCR Systems. Primer sequences are listed in Table A2. β-Actin served as the internal control, and relative expression levels were calculated with the 2^−∆∆CT^ method [21].

### 2.9. Colon Transcriptome Sequencing

Total RNA was extracted and assessed by agarose gel electrophoresis and Nanodrop spectrophotometer. Poly(A) mRNA was isolated to construct cDNA libraries via end repair, adapter ligation, and PCR amplification. Sequencing was performed on the Illumina HiSeq platform. SOAPnuke was used to filter reads containing adapters, low-quality bases, or excessive ambiguous bases. Differentially expressed genes (DEGs) were identified using DESeq2 (|log_2_FC| ≥ 1 and *p*, FDR < 0.05). Gene Ontology (GO) and Kyoto Encyclopedia of Genes and Genomes (KEGG) enrichment analyses were conducted using the Dr. Tom multi-omics analysis platform (https://biosys.bgi.com).

### 2.10. Gut Microbiota 16S rRNA Gene Sequencing

Fecal samples collected at the end of the modeling period were used for 16S rRNA microbial analysis. Microbial profiling was carried out by Majorbio Bio-Pharm Technology Co., Ltd. (Shanghai, China). Fecal bacterial DNA was isolated using the E.Z.N.A. Soil DNA Kit (Omega Bio-tek, Norcross, GA, USA). The V3-V4 region of 16S rRNA genes was amplified with barcoded primers (338F, ACTCCTACGGGAGGCAGCAG; 806R, ACTCCTACGGGAGGCAGCAG). Sequencing was performed on the Illumina MiSeq platform, and downstream analyses were conducted using the Majorbio Cloud Platform (https://cloud.majorbio.com).

### 2.11. Statistical Analysis

All values are presented as mean ± standard deviation (SD). Statistical significance among groups was determined using one-way ANOVA followed by Tukey’s post hoc test using GraphPad Prism software (version 10.1.2). *p*-value < 0.05 was considered statistically significant.

## 3. Results

### 3.1. L. plantarum 001 Exhibits Potent Anti-Inflammatory Properties In Vitro

**Figure 1:**
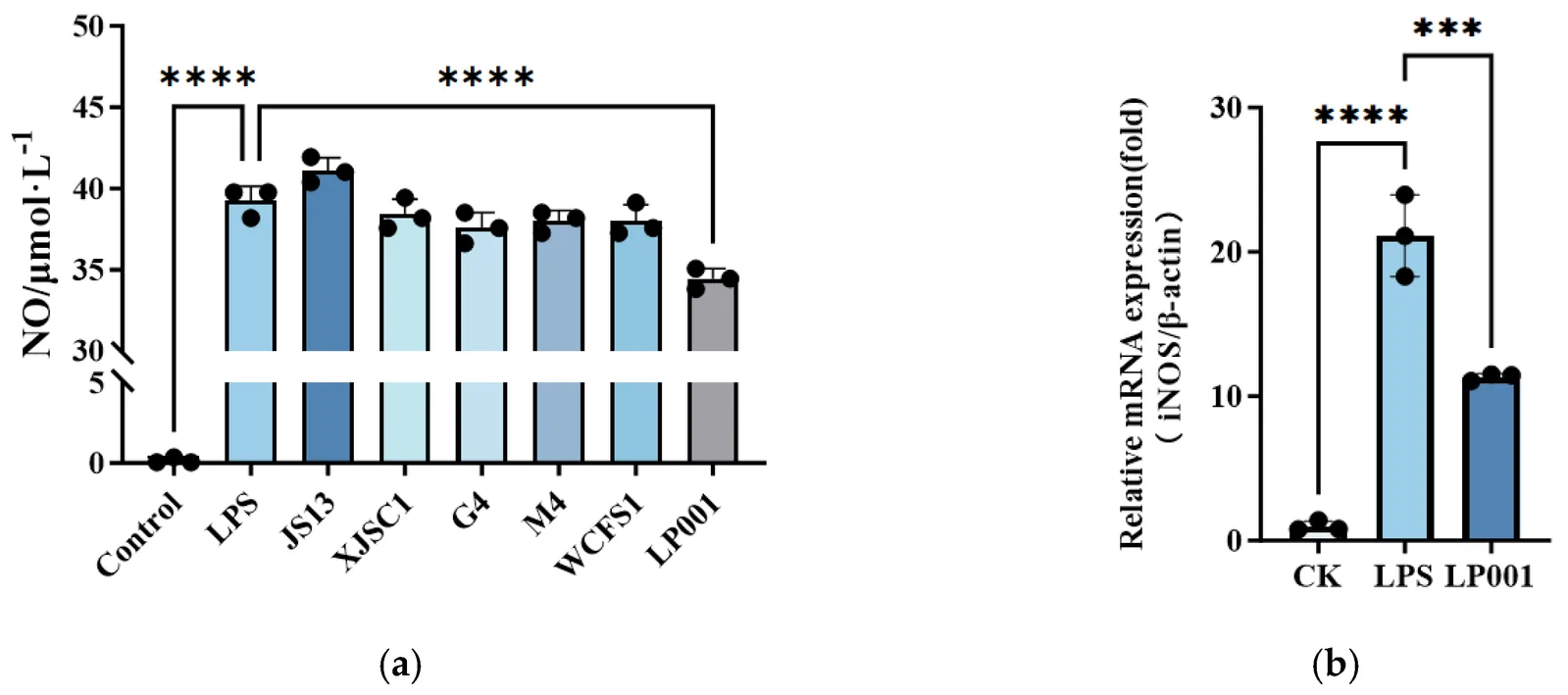

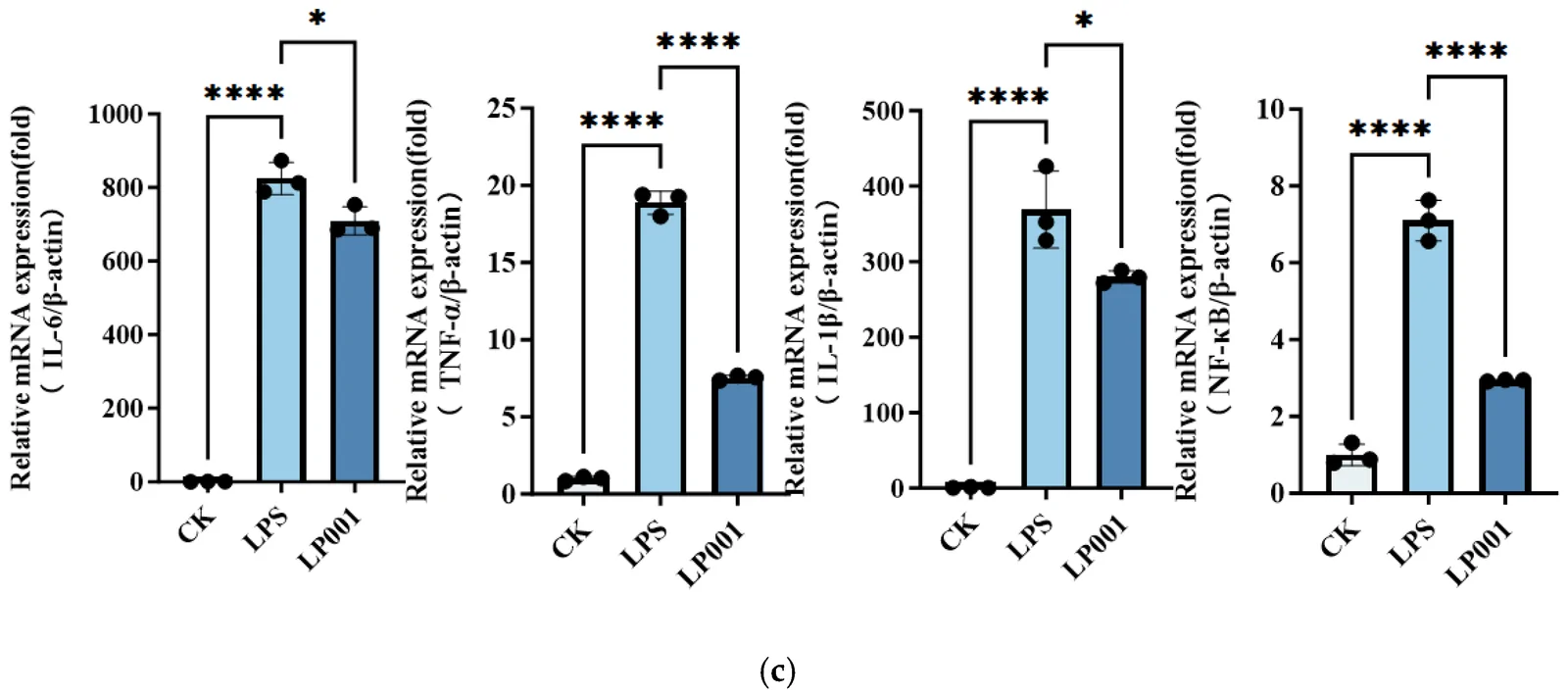
Anti−inflammatory effects of *L. plantarum* strains in LPS−stimulated RAW 264.7 Macrophages. (**a**) Nitric oxide (NO) inhibition by six *L. plantarum* strains. (**b**) Relative mRNA expression of inducible nitric oxide synthase (*iNOS*) in inflammatory RAW264.7 cells. (**c**) Relative mRNA expression of pro-inflammatory cytokines (*IL-6*, *TNF-α*, *IL-1β*, *NF-κB*) in inflammatory RAW264.7 cells. Data were presented as mean ± SD. CK represents the control group, LPS represents the LPS-treated group and LP001 represents the *L. plantarum* 001-treated group. *P*-values were determined by one-way ANOVA followed by Tukey’s test. * *p* < 0.05, *** *p* < 0.001, and **** *p* < 0.0001.

The anti-inflammatory potential of six *L. plantarum* strains was evaluated using an LPS−stimulated RAW264.7 macrophage model. As shown in Figure 1a, compared with LP group, only *L. plantarum* 001 treatment significantly reduced NO production. Furthermore, compared with the control group, *iNOS* expression was upregulated 21-fold in LPS group. However, the invention with *L. plantarum* 001 significantly reduced the level of *iNOS* expression to 53.72% (Figure 1b). In addition, the mRNA levels of *IL-6*, *TNF-α*, and *IL-1β* was upregulated 825.1-fold, 18.89-fold and 369.1-fold in response to LPS group, respectively. Intervention with *L. plantarum* 001 significantly attenuated the expression of all three cytokines (Figure 1c). It is worth noting that *NF-κB* expression was up-regulated by 7-fold in the LPS group and then reduced to 41.40% after *L. plantarum* 001 treatment. Therefore, *L. plantarum* 001 was selected for subsequent in vivo evaluation of its potential to alleviate IBS symptoms.

### 3.2. L. plantarum 001 Attenuates IBS Related Symptoms

**Figure 2:**
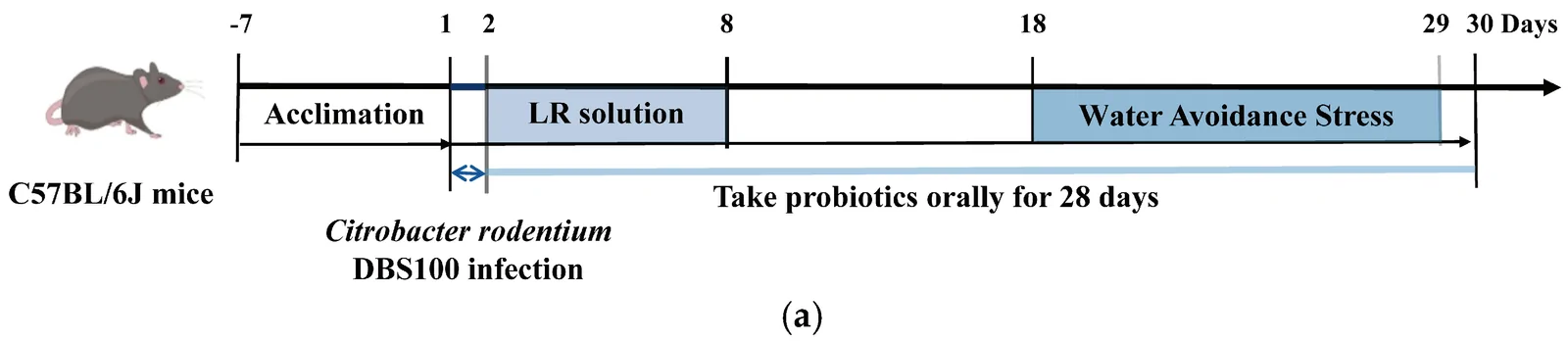

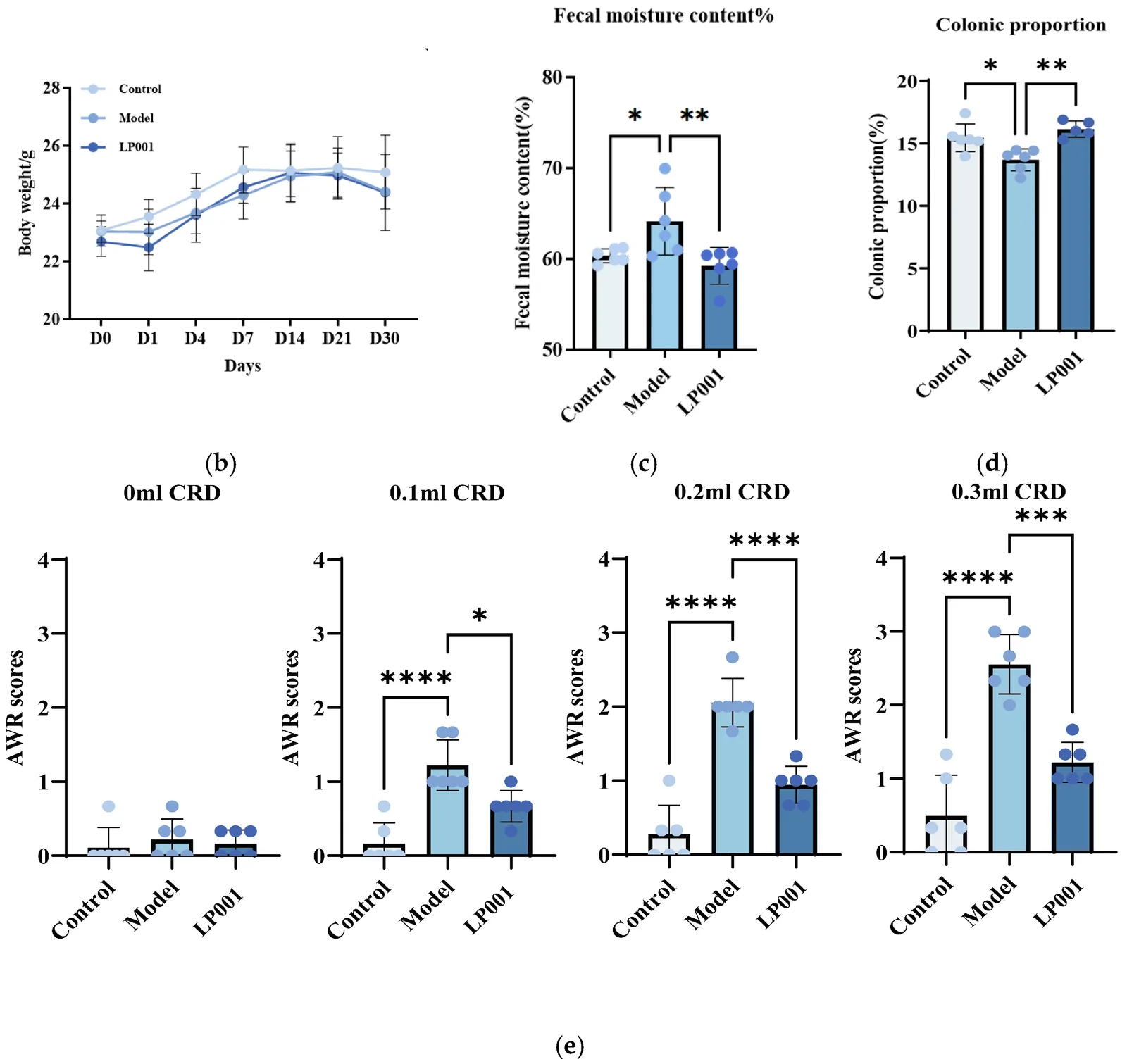
Animal experimental design and evaluation of the alleviating effect of *L. plantarum* 001 on irritable bowel syndrome. (**a**) Animal Experiment Design. (**b**) Body weight changes during modeling period in each group of mice. (**c**) Water content in feces. (**d**) Proportion of colon to the entire intestine. (**e**) Comparison of the abdominal withdrawal reflex (AWR) scores of different groups. The LR solution used in the experiment refers to lactated Ringer’s solution, and “model” denotes the IBS model group. *n* = 6 in each group. Data were presented as mean ± SD. *p*-values were determined by one-way ANOVA followed by Tukey’s test. * *p* < 0.05, ** *p* < 0.01, *** *p* < 0.001, and **** *p* < 0.0001.

The experiment was designed to assess the alleviative effects of *L. plantarum* 001 on IBS in C57BL/6J illustrated in Figure 2a. The results showed that there was no significant difference in body weight among the three groups (Figure 2b). Compared with the control group, the fecal moisture content was significantly increased to 64.16 ± 3.71% in the model group. *L. plantarum* 001 intervention significantly reduced the fecal moisture to 59.24 ± 2.02% (Figure 2c). Compared with the control group, the proportion of the colon to the total intestine was significantly decreased to 13.69 ± 0.88% in the model group. It is worth noting that *L. plantarum* 001 intervention significantly increased the colon proportion to 16.14 ± 0.65% (Figure 2d). Furthermore, the AWRs was significantly increased in the model group compared with the control group. Notably, *L. plantarum* 001 treatment significantly reduced AWRs at gas infusion volumes of 0.1, 0.2, and 0.3 mL, indicating attenuation of visceral hypersensitivity (Figure 2e).

### 3.3. L. plantarum 001 Improved Intestinal Barrier Damage in IBS

**Figure 3:**
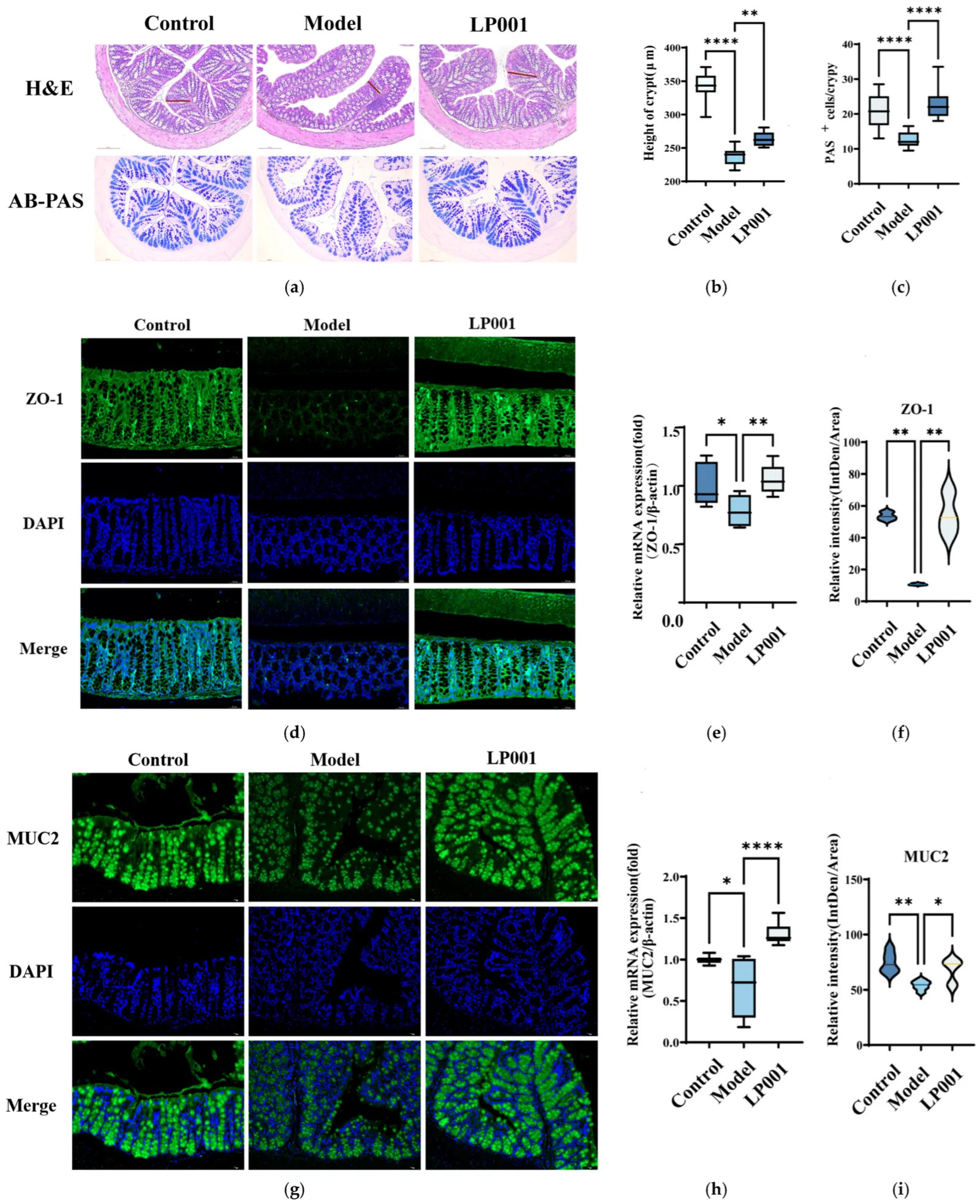
The effect of *L. plantarum* 001 on colon tissue of mice with irritable bowel syndrome. (**a**) H&E staining and AB-PAS staining images of colon tissues. The crypt height in panel (**a**) is indicated by a red line. Scale bar means 200 μm. (**b**) Height of crypt. Crypt height was quantified from six mice per group, with ten well-oriented crypts selected from each mouse. (**c**) goblet cells counting per crypt. For each group, two AB-PAS-stained images were analyzed, and twenty crypts per image were counted for goblet cell quantification. (**d**) Immunofluorescence staining images of ZO-1 (green). Nuclei were stained by DAPI (blue). Scale bar means 50 μm. (**e**) Relative mRNA expression of *ZO-1* gene in colonic tissues normalized by β-actin. (**f**) Quantification of ZO-1 fluorescence intensity. (**g**) Immunofluorescence staining images of MUC-2 (green). Nuclei were stained by DAPI (blue). Scale bar means 50 μm. (**h**) Relative mRNA expression of *MUC-2* gene in colonic tissues normalized by β-actin. (**i**) Quantification of MUC-2 fluorescence intensity. Data were presented as mean ± SD. *P*-values were determined by one-way ANOVA followed by Tukey’s test. * *p* < 0.05, ** *p* < 0.01, and **** *p* < 0.0001.

H&E staining showed that inflammatory cell infiltration was observed in the model group (Figure 3a). Crypt depth was also significantly decreased from 320.4 ± 20.46 μm in the control group to 237.5 ± 12.89 μm in the model group. *L. plantarum* 001 intervention significantly increased crypt depth to 263.7 ± 11.10 μm (Figure 3b). Furthermore, the number of goblet cells in the colon was 12 cells per crypt in the model group, which was significantly lower than that of control group. However, *L. plantarum* 001 intervention increased the number of goblet cells to 22 cells per crypt (Figure 3c). In addition, compared with the control group, the expression of *ZO-1* and *MUC2* in the colon tissue was significantly downregulated at mRNA level in the model group (Figure 3e,h). Treatment with *L. plantarum* 001 upregulated *ZO-1* and *MUC2* expression by 1.04-fold and 1.20-fold, respectively, compared with the model group. Immunofluorescence further confirmed that the expression of ZO-1 and MUC2 was downregulated in the model group, and *L. plantarum* 001 intervention significantly restored the expression level of ZO-1 and MUC-2 to that of the control group (Figure 3d,f). Together, these findings indicated that *L. plantarum* 001 ameliorates intestinal barrier damage in IBS mice by restoring crypt architecture, enhancing goblet cell abundance, and increasing the expression of key barrier-associated proteins.

### 3.4. L. plantarum 001 Exhibited Anti-Inflammatory Effects to Alleviate IBS via Transcriptome Sequencing

**Figure 4:**
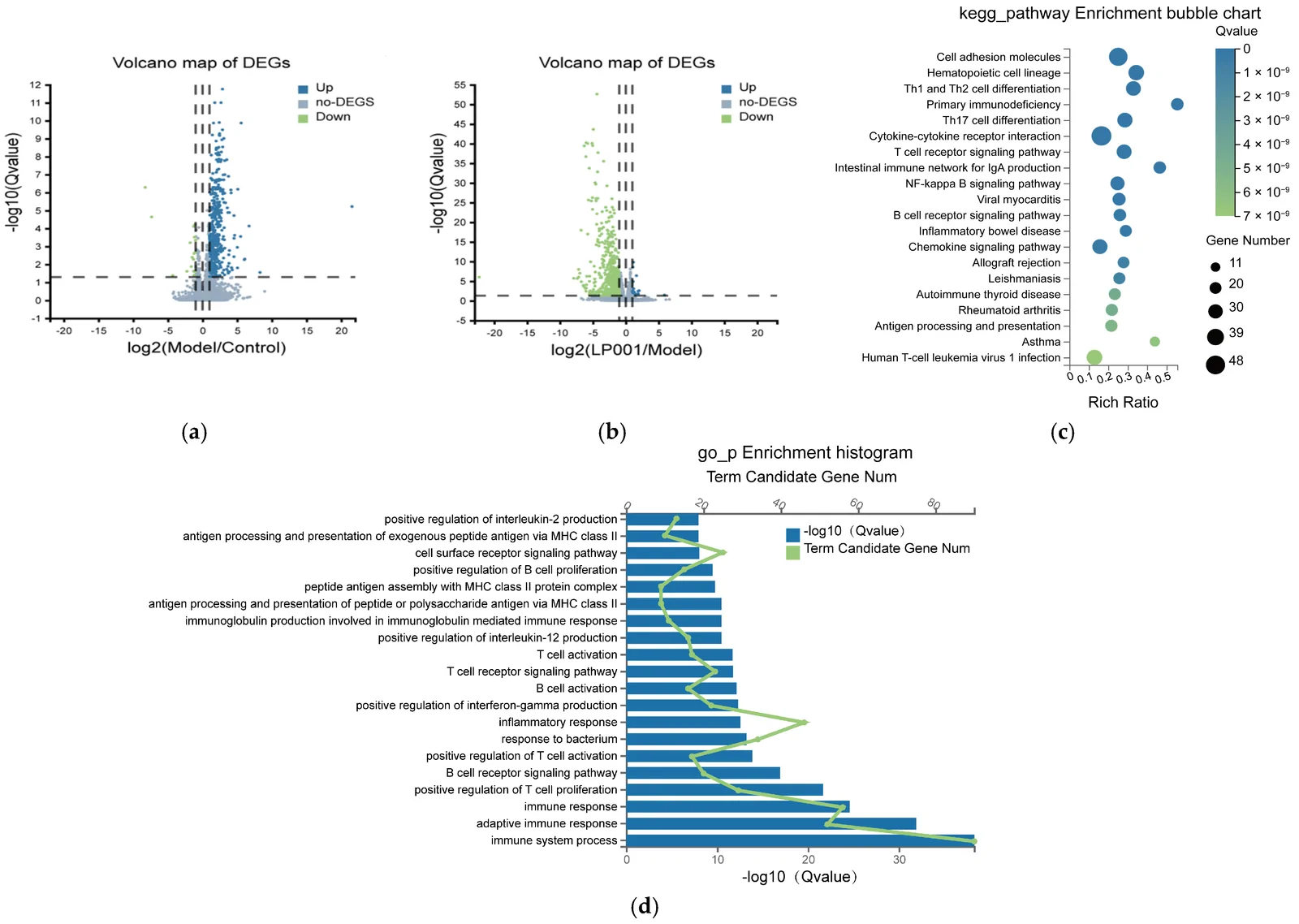

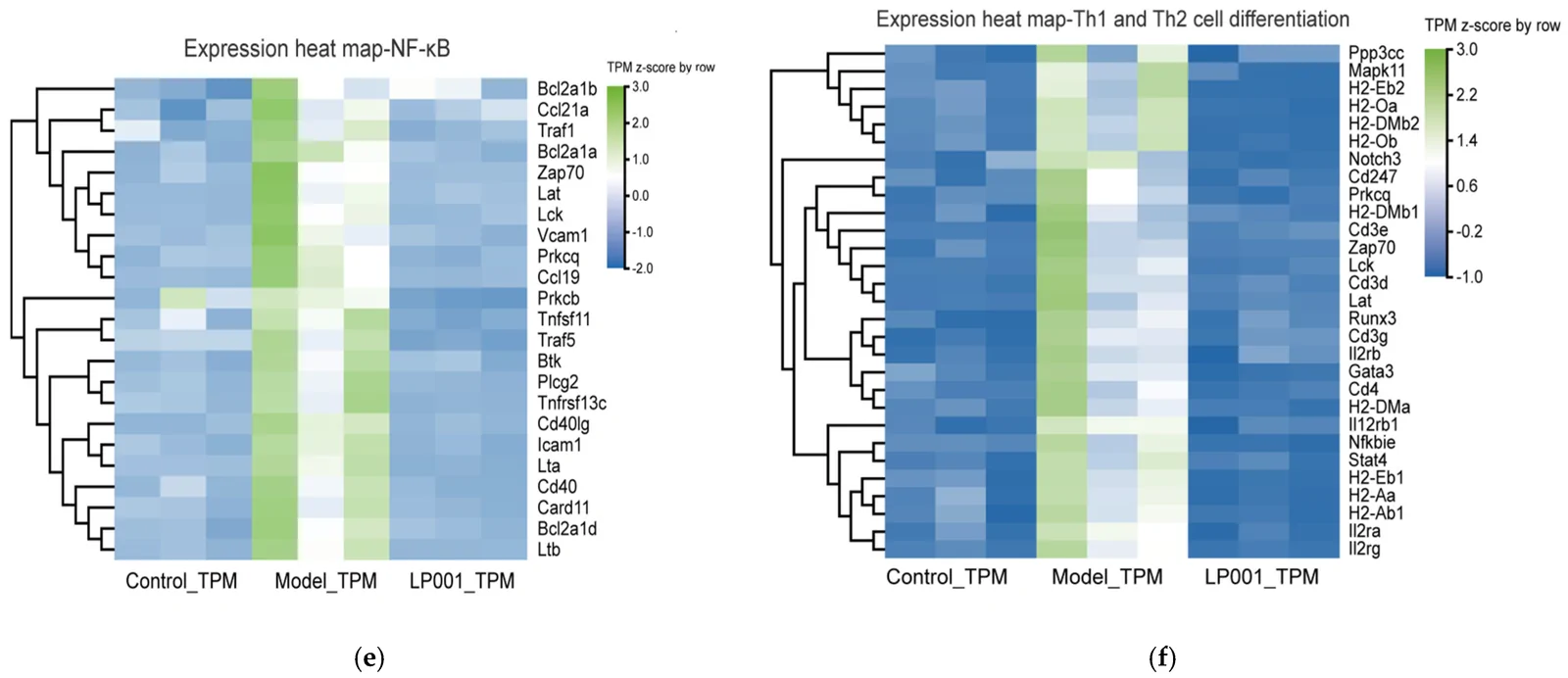
Transcriptomic analysis of *L. plantarum* 001 on irritable bowel syndrome. (**a**) Volcano plot comparing Model and Control groups. (**b**) Volcano plot comparing *L. plantarum* 001 and Model groups. (**c**) KEGG enrichment analysis. (**d**) GO enrichment analysis. (**e**) Expression heat map of NF-κB signaling pathway. (**f**) Expression heat map of Th1 and Th2 cell differentiation. All transcriptomic analyses were performed using colon tissue samples.

In order to explore the underlying mechanism by which *L. plantarum* 001 alleviates IBS, transcriptome sequencing of the colon was performed in this study. Differential expression analysis revealed 452 genes exhibiting significant transcriptional changes between the IBS and control groups, comprising 434 upregulated genes and 18 downregulated genes (FDR < 0.05, |log_2_FC| ≥ 1). Comparison of the LP001-treated group with the IBS group revealed 591 differentially expressed genes, of which 576 were downregulated and 15 were upregulated (Figure 4a,b).

GO enrichment analysis showed that these genes were significantly enriched in immune and inflammation. These processes mainly included immune regulation (regulation of immune system, adaptive immune response, and innate immune response), cell activation (activation of T cells and B cells and their receptor signaling pathways), and regulation of inflammatory responses (Figure 4c,d). Furthermore, the DEGs were significantly enriched in immune cell differentiation including Th1/Th2 cell differentiation pathway and Th17 cell differentiation pathway, signal transduction including T cell receptor signaling pathway and NF-κB signaling pathway, and regulation of inflammation including cytokine-cytokine receptor interaction pathway (Figure 4e,f). Further analysis revealed that 23 genes in the NF-κB signaling pathway and 29 genes in the Th1/Th2 differentiation pathway were significantly upregulated in the model group compared to the control (Table A3 and Table A4). However, *L. plantarum* 001 intervention restored these gene expression levels to near-normal levels, demonstrating its potential in modulating immune and inflammatory pathways to alleviate IBS symptoms.

### 3.5. L. plantarum 001 Alleviated IBS by Modulating the NF-κB Signaling Pathway

**Figure 5:**
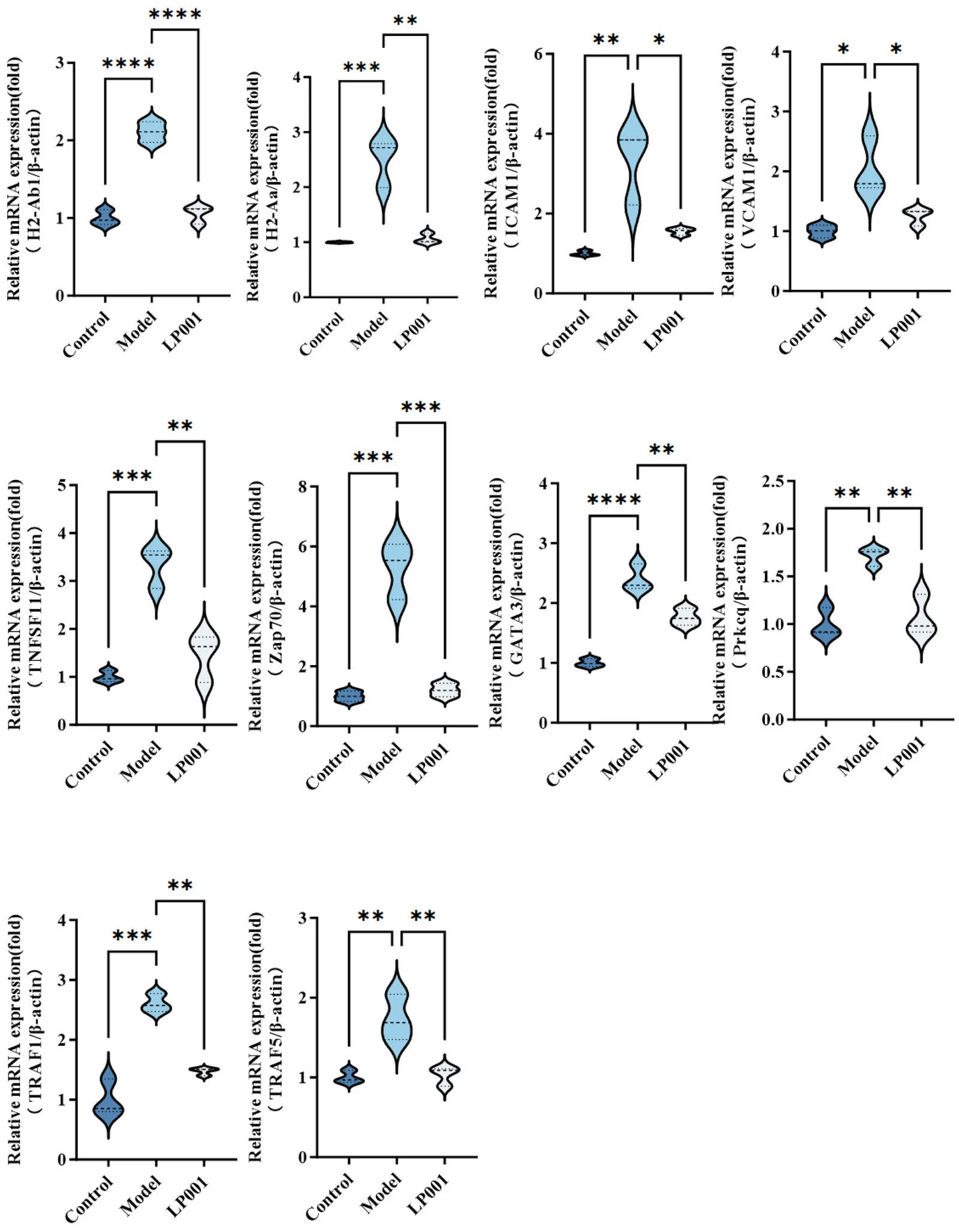
Relative mRNA expression levels of genes in the NF-κB signaling pathway and Th1/Th2 cell differentiation. All analyses were conducted using colon tissue samples. Data were presented as mean ± SD. *P*-values were determined by one-way ANOVA followed by Tukey’s test. * *p* < 0.05, ** *p* < 0.01, *** *p* < 0.001, and **** *p* < 0.0001.

The expression levels of genes involved in Th1/Th2 cell differentiation and NF-κB signaling pathway were further confirmed by RT-qPCR (Figure 5). Compared with the control group, the expression levels of Tnfsf11, Traf1, Traf5, and Prkcq were upregulated 3.3-fold, 2.6-fold, 1.7-fold, and 1.7-fold in the model group, respectively. Zap70, a crucial player in T cell receptor signaling, was upregulated 5.2-fold in the model group. In addition, Vcam1 and Icam1 encoding effector molecules were upregulated 2.0-fold and 3.3-fold in the model group, which are involved in immune cell adhesion and migration. Then the transcription factor Gata3 was upregulated 2.4-fold in the model group, which regulates Th17 cell differentiation. MHC class II molecules H2-Aa and H2-Ab1 were, respectively, upregulated 2.4-fold and 2.1-fold in the model group, indicating it could enhance antigen presentation and T cell activation. However, *L. plantarum* 001 intervention almost restored the mRNA levels of all these genes to that of the control group mice. The RT-qPCR results were consistent with the transcriptome analysis, confirming that *L. plantarum* 001 downregulated genes associated with the NF-κB signaling pathway and Th1/Th2 differentiation. This intervention thus inhibited abnormal immune activation associated with IBS and contributed to the therapeutic effects of *L. plantarum* 001.

### 3.6. L. plantarum 001 Reshaped Gut Microbiota Balance to Alleviate IBS

**Figure 6:**
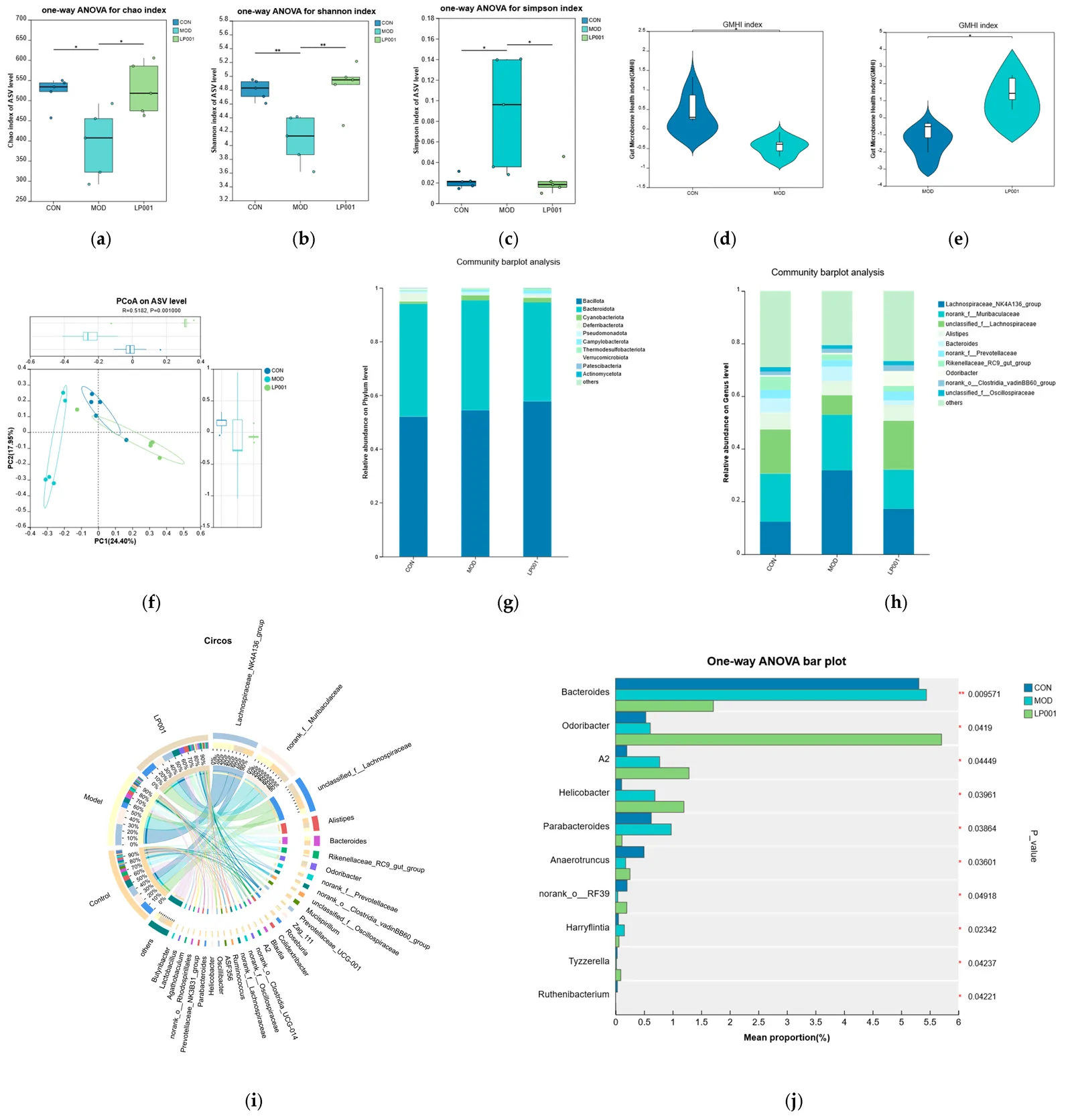
Effects of *L. plantarum* 001 on gut microbiota composition in IBS mice. (**a**) Chao index of Alpha diversity. (**b**) Shannon index of Alpha diversity. (**c**) Simpson index of Alpha diversity. (**d**,**e**) Gut microbiome health index (GMHI). (**f**) Principal coordinates analysis (PCoA) plot on ASV level of microbiota. (**g**) Bar diagram of community at the Phylum level. (**h**) Bar diagram of community at the Genus level. (**i**) Circos diagram of community at the Genus level. (**j**) Diversity map of community species abundance at the Genus level. * *p* < 0.05, ** *p* < 0.01.

To explore the effects of *L. plantarum* 001 on the gut microbiota of mice with IBS, 16S rRNA sequencing was performed in this study. α-diversity analysis showed that the Chao index and Shannon index were significantly reduced, while the Simpson index was significantly increased in the model group (Figure 6a–c). β-diversity analysis exhibited a significant difference between the control and model groups on ASV level (PC1 = 24.40%, PC2 = 17.95%, ANOSIM *p* < 0.05). A segregation was also observed between the microbiota structure of LP001 group and that of the model group (Figure 6f). This finding indicates that IBS leads to impaired ecological complexity of the flora and probiotics can reshape the disrupted intestinal microbiota. Furthermore, the GMHI of the model group was significantly lower than that of the control group (*p* < 0.05), After intervention with *L. plantarum* 001, the GMHI significantly increased (*p* < 0.05) (Figure 6d,e).

At the phylum level, *Bacillota* and *Bacteroidota* were the predominant phyla in fecal microbiota samples (Figure 6g). The relative abundance of *Deferribacterota* decreased from 3% in the control group to 0.1% in the model group. This phylum includes genera with mucosal repair functions (such as *Mucispirillum*), and its depletion is directly associated with intestinal barrier damage in IBS. And the abundance of *Pseudomonadota* significantly increased from 0.7% in the control group to 1.1%. This phylum encompasses a variety of Gram-negative pathogens (such as *Enterobacteriaceae*), and its excessive proliferation can release endotoxin (e.g., LPS), driving low-grade intestinal inflammation. However, intervention with *L. plantarum* 001 effectively reversed the abundance of *Pseudomonadota*. The abundance of *Desulfobacteria* phylum recovered to 2.5% and the abundance of *Pseudomonadophyta* was reversed to 0.8%. These results confirmed that probiotics can reshape the microecological balance of the IBS gut by inhibiting the expansion of pathogenic bacteria and restoring protective microbiota. Notably, at the genus level, *Odoribacter* showed significant differences among the three groups and was significantly enriched in the LP group (Figure 6h–j). This genus can produce short-chain fatty acids (such as butyric acid), which have anti-inflammatory and intestinal barrier protective effects.

## 4. Discussion

Irritable bowel syndrome (IBS) imposes considerable socioeconomic burdens due to its chronic symptoms and significant impact on quality of life [4,22]. The pathogenesis of IBS involves environmental triggers such as enteric infections and psychological stress, along with mucosal immune activation, low-grade inflammation, epithelial barrier dysfunction, and visceral hypersensitivity [2,3]. In this study, an IBS mouse model was generated through the combined use of *Citrobacter rodentium* infection and water avoidance stress. This induction strategy produced characteristic features of low-grade intestinal inflammation, including modest inflammatory cell infiltration, elevated pro-inflammatory cytokine expression, and compromised epithelial barrier integrity. These results are consistent with the clinical symptoms of low-grade IBS-related inflammation [23,24]. In addition, mice in the model group developed diarrhea and pronounced visceral hypersensitivity, a defining pathophysiological feature of IBS characterized by heightened pain responses to visceral stimuli [25,26,27]. These results confirmed that *C. rodentium* combined with water avoidance stress provides a reliable approach for simulating IBS pathogenesis in mice. Notably, intervention with *L. plantarum* 001 ameliorated diarrheal phenotypes, restored colonic mucosal architecture and attenuated visceral hypersensitivity, indicating its therapeutic promise for mitigating IBS-related disturbances.

Dysregulation of the intestinal barrier represents a central pathological attribute of IBS, evidenced by heightened epithelial permeability, downregulated tight junction components, and a compromised mucus barrier [24]. ZO-1 serves as a critical structural component of tight junctions, and reduced levels of this protein in IBS patients have been closely linked to compromised epithelial barrier integrity and increased symptom severity [24,28]. In our study, *L. plantarum* 001 intervention significantly restored colonic ZO-1 expression. Similar observations have been reported for *L. plantarum* MB452, which improves tight junction function by upregulating genes involved in junctional assembly [29,30]. Restoring ZO-1 is thought to limit the translocation of pathogen-associated molecular patterns such as lipopolysaccharide across the mucosal barrier, thereby dampening downstream TLR4-driven inflammatory responses [28]. Furthermore, *L. plantarum* 001 significantly increased goblet cell numbers and upregulated MUC2 gene expression and protein levels in this study. Enhanced mucin secretion thickens the mucus layer, which creates a physical barrier that limits bacterial-epithelial contact and provides glycans for commensal maintenance [31]. These findings suggest that *L. plantarum* 001 alleviates IBS-related symptoms by protecting epithelial structure and function, thereby preventing excessive microbial translocation and subsequent mucosal immune activation.

In addition to these barrier-protective effects, *L. plantarum* 001 also exerted potent immunomodulatory activity by targeting the NF-κB signaling pathway, a central driver of chronic low-grade inflammation in IBS. Hyperactivation of NF-κB is known to drive the overproduction of pro-inflammatory cytokines, compromise epithelial barrier integrity, and contribute to the development of visceral hypersensitivity [17,18]. It has been reported that TRAF1/5 are critical adaptor proteins that mediate NF-κB activation by recruiting downstream signaling molecules, while PRKCQ modulates NF-κB transduction through phosphorylation-dependent regulation of IκB kinase (IKK) complex. In this study, *L. plantarum* 001 significantly downregulated the expression of key upstream regulators (*TRAF1*, *TRAF5*) and kinase modulators (*PRKCQ*) of the NF-κB pathway to suppress NF-κB activation. In addition, *L. plantarum* 001 normalized NF-κB-associated Th1/Th2 dysregulation by restoring T-cell receptor signaling balance (*Zap70*), repressing Th2 lineage specification (*Gata3*), and modulating cytokine responsiveness, thereby mitigating Th1-derived IFN-γ-induced barrier compromise [32]. Furthermore, *L. plantarum* 001 reduced expression of adhesion molecules *Vcam1* and *Icam1* by inhibiting the NF-κB/Th axis, thereby reducing leukocyte recruitment implicated in IBS mucosal immunopathology [33]. These processes blocked the key inflammatory cascade in the pathogenesis of IBS.

Intestinal microbiota dysbiosis is increasingly recognized as a central pathogenic mechanism in IBS, characterized by reduced microbial diversity, loss of beneficial bacteria, and expansion of potential pathogenic bacteria [7,8]. In this study, the model group exhibited an elevated proportion of *Pseudomonadota*, which is a phylum enriched with pro-inflammatory taxa [7]. Clinically, *Pseudomonadota* overgrowth in IBS-D patients correlates with bile acid malabsorption and rapid colonic transit, both of which contribute to diarrheal symptoms [34,35]. *L. plantarum* 001 reduced *Pseudomonadota* abundance to potentially alleviate intestinal inflammation. In addition, *L. plantarum* 001 increased the abundance of *Odoribacter*, which is a recognized butyrate producer [36,37]. Butyrate, a major short-chain fatty acid (SCFA), plays dual protective roles in IBS. It strengthens epithelial barrier integrity by promoting mucin production and enhancing the expression of tight junction proteins [29,30], and it also mitigates inflammation by inhibiting histone deacetylases (HDACs) and reducing the synthesis of pro-inflammatory cytokines [36,38]. Notably, butyrate-producing bacteria including *Odoribacter* have also been reduced in the mucosa-associated microbiota of IBS and inflammatory bowel disease (IBD) patient [37]. Taken together, the intervention with *L. plantarum* 001 improved barrier integrity, attenuated mucosal inflammation, and reshaped gut microbial composition, collectively contributing to its beneficial effects on IBS-related symptoms.

It is worth noting that the changes in fecal moisture observed in this study were relatively modest, even though statistical significance was achieved. Similar mild alterations have been documented in IBS, especially in diarrhea-predominant presentations, where disturbances in bile acid metabolism, epithelial permeability and mucosal function tend to result in subtle shifts in fecal water content rather than pronounced dehydration or fluid loss. Therefore, the biological relevance of this parameter should be interpreted cautiously, and it may reflect the mild diarrheal phenotype characteristic of low-grade IBS rather than a strong secretory response.

Several molecular mechanisms may contribute to the anti-inflammatory effects observed with *L. plantarum* 001. Previous studies have shown that *L. plantarum* strains can produce metabolites, such as SCFAs, that enhance epithelial barrier integrity and promote goblet cell function [29,30,36,37,38]. The increase in butyrate-producing bacteria observed in our study is consistent with this mechanism. In addition, the reduction in *Pseudomonadota*, a phylum associated with pro-inflammatory activity and accelerated transit in IBS-D [34,35] suggests that competitive exclusion or ecological reshaping of the microbiota may contribute to the anti-inflammatory environment. *L. plantarum* strains have also been shown to modulate innate immune pathways, particularly by dampening TLR-mediated NF-κB activation, which aligns with our transcriptomic and RT-qPCR findings of reduced TRAF1/5 and PRKCQ expression.

Although this study highlights the multifaceted protective actions of *L. plantarum* 001 in an IBS model, several important questions remain. The mechanistic basis for its strain-specific activity is still not fully resolved. Our data suggest concurrent improvements in epithelial barrier function, inflammatory signaling, and microbial composition, yet the specific metabolites and host pathways mediating these effects require further clarification. Approaches such as metabolomics or controlled single-strain colonization may help disentangle these contributions. Additionally, the present work employed an acute IBS model driven by *C. rodentium* infection and stress. Whether *L. plantarum* 001 confers similar benefits in chronic or alternative models remains to be determined. The enrichment of butyrate-producing taxa, including *Odoribacter*, is a notable finding, but its causal relevance to symptom improvement should be validated through targeted depletion or transplantation studies. Finally, translating these findings to human populations will require careful evaluation. Variations in baseline microbiota, diet, and immune profiles between mice and humans may substantially influence probiotic responses. Well-designed clinical trials, together with long-term safety assessments, are therefore essential to establish whether *L. plantarum* 001 can be advanced as a reliable therapeutic option for IBS. Continued mechanistic and translational research will be critical for defining its full therapeutic potential.

## 5. Conclusions

In this study, we established an IBS mouse model using *Citrobacter rodentium* infection combined with water avoidance stress and demonstrated that *L. plantarum* 001 ameliorates IBS pathology through a multi-targeted mechanism. *L. plantarum* 001 restored the intestine barrier integrity by upregulating ZO-1 and MUC2, attenuated chronic inflammation by inhibiting NF-κB signaling and normalizing Th1/Th2 immune responses, and reshaped the gut microbiota by enriching beneficial butyrate-producing taxa while suppressing pro-inflammatory *Pseudomonadota*. This coordinated triad of actions provides a robust molecular basis for the use of microbial interventions in IBS management and highlights *L. plantarum* 001 as a promising probiotic candidate for adjunctive therapy.

## Data Availability

The original contributions presented in this study are included in the article. The raw RNA-seq data have been deposited in the NCBI Sequence Read Archive (SRA) under accession number PRJNA1369013. Further inquiries can be directed to the corresponding authors.

## Abbreviations

The following abbreviations are used throughout this manuscript:

IBS	Irritable bowel syndrome
FGID	Functional Gastrointestinal Disorder
FMT	Fecal Microbiota Transplant
*L. plantarum*	*Lactiplantibacillus plantarum*
NF-κB	Nuclear Factor-κB
LPS	Lipopolysaccharides
NO	Nitric Oxide
IL-1β	Interleukin-1β
COX-2	Cyclooxygenase-2
iNOS	Inducible nitric oxide synthase
TNF-α	Tumor necrosis factor-alpha
IL-6	Interleukin-6
VH	Visceral hypersensitivity
SD	Standard Deviations
WAS	Water avoidance stress
CRD	Colorectal distension
AWRs	Abdominal withdrawal reflexes Scores
SPF	Specific pathogen free
PBS	Phosphate-buffered saline
ddH2O	Double distilled water
CFU	Colony-forming unit
H&E	Hematoxylin-eosin staining
AB-PAS	Alcian blue-periodic acid-Schiff
PCR	Polymerase chain reaction
RT-PCR	Reverse transcription-PCR
RT-qPCR	Real time quantitative PCR
TJ	Tight junction
KEGG	Kyoto Encyclopedia of Genes and Genomes
GO	Gene Ontology
DEGs	Differentially expressed genes
GMHI	Gut Microbiome Health Index
PCoA	Principal coordinate analysis
LDA	Linear discriminant analysis
SCFAs	Short-chain fatty acids
ASV	Amplicon sequence variant
HPLC	High Performance Liquid Chromatography

## Appendix A

**Table A1:** Strains used in this work.

Strain Name	Latin Name	Strain Source
JS13	*Lactiplantibacillus plantarum*	Jiangshui
XJSC1	*Lactiplantibacillus plantarum*	Pickles vegetables
G4	*Lactiplantibacillus plantarum*	Koumiss
M4	*Lactiplantibacillus plantarum*	Milk
WCFS1	*Lactiplantibacillus plantarum*	Saliva-human oral cavity
LPPerfectus001	*Lactiplantibacillus plantarum*	Pickles vegetables

**Table A2:** List of RT-qPCR primers applied for gene expression analysis in this study.

Gene	Forward Primer (5′-3′)	Reverse Primer (5′-3′)
*β-actin*	GTGACGTTGACATCCGTAAAGA	GCCGGACTCATCGTACTCC
*Tnf-α*	CCTGTAGCCCACGTCGTAG	GGGAGTAGACAAGGTACAACCC
*Il-6*	CCAAGAGGTGAGTGCTTCCC	CTGTTGTTCAGACTCTCTCCCT
*COX-2*	TTCAACACACTCTATCACTGGC	AGAAGCGTTTGCGGTACTCAT
*i-NOS*	ACCCAAGGTCTACGTTCAGG	CGCACATCTCCGCAAATGTA
*NF-κB*	ATGGCAGACGATGATCCCTAC	TGTTGACAGTGGTATTTCTGGTG
*ZO-1*	GGCCTTGGCCTAGCATACAC	GTCTTCATTTGACCCTCCCTC
*Muc2*	TGCTGACGAGTGGTTGGTGAAT	GATGAGGTGGCAGACAGGAGAC
*H2-Ab1*	AGCCCCATCACTGTGGAGT	GATGCCGCTCAACATCTTGC
*H2-Aa*	TCAGTCGCAGACGGTGTTTAT	GGGGGCTGGAATCTCAGGT
*Icam1*	GTGATGCTCAGGTATCCATCCA	CACAGTTCTCAAAGCACAGCG
*Vcam1*	AGTTGGGGATTCGGTTGTTCT	CCCCTCATTCCTTACCACCC
*Tnfsf11*	CAGCATCGCTCTGTTCCTGTA	CTGCGTTTTCATGGAGTCTCA
*Zap70*	CTACGTGCTGTCGTTGGTG	GTTACACGGCTTACGCAGGT
*Gata3*	CTCGGCCATTCGTACATGGAA	GGATACCTCTGCACCGTAGC
*Prkcq*	TCCGCCAGCATCCTTTGTTT	TCCTTGTCGAAATTGCTACAGTC
*Traf1*	AGGGTGGTGGAATTACAGCAA	GCAGTGTAGAAAGCTGGAGAG
*Traf5*	TTTGAGCCCGACACCGAGTA	AGAGACCGGATGCACTGCT

**Table A3:** The differentially expressed genes in the NF-κB signaling pathway.

Gene ID	Gene	Gene Description
108723	*Card11*	caspase recruitment domain family, member 11
12044	*Bcl2a1a*	B cell leukemia/lymphoma 2 related protein A1a
12045	*Bcl2a1b*	B cell leukemia/lymphoma 2 related protein A1b
12047	*Bcl2a1d*	B cell leukemia/lymphoma 2 related protein A1d
12229	*Btk*	Bruton agammaglobulinemia tyrosine kinase
15894	*Icam1*	intercellular adhesion molecule 1
16797	*Lat*	linker for activation of T cells
16818	*Lck*	lymphocyte protein tyrosine kinase
16992	*Lta*	lymphotoxin A
16994	*Ltb*	lymphotoxin B
18751	*Prkcb*	protein kinase C, beta
18761	*Prkcq*	protein kinase C, theta
18829	*Ccl21a*	C-C motif chemokine ligand 21 (serine)
21939	*Cd40*	CD40 antigen
21943	*Tnfsf11*	tumor necrosis factor (ligand) superfamily, member 11
21947	*Cd40lg*	CD40 ligand
22029	*Traf1*	TNF receptor-associated factor 1
22033	*Traf5*	TNF receptor-associated factor 5
22329	*Vcam1*	vascular cell adhesion molecule 1
22637	*Zap70*	zeta-chain (TCR) associated protein kinase
234779	*Plcg2*	phospholipase C, gamma 2
24047	*Ccl19*	C-C motif chemokine ligand 19
72049	*Tnfrsf13c*	tumor necrosis factor receptor superfamily, member 13c

**Table A4:** The differentially expressed genes in the Th1/Th2 differentiation pathway.

Gene ID	Gene	Gene Description
12399	*Runx3*	runt related transcription factor 3
12500	*Cd3d*	CD3 antigen, delta polypeptide
12501	*Cd3e*	CD3 antigen, epsilon polypeptide
12502	*Cd3g*	CD3 antigen, gamma polypeptide
12503	*Cd247*	CD247 antigen
12504	*Cd4*	CD4 antigen
14462	*Gata3*	GATA binding protein 3
14960	*H2-Aa*	histocompatibility 2, class II antigen A, alpha
14961	*H2-Ab1*	histocompatibility 2, class II antigen A, beta 1
14969	*H2-Eb1*	histocompatibility 2, class II antigen E beta
14998	*H2-DMa*	histocompatibility 2, class II, locus DMa
14999	*H2-DMb1*	histocompatibility 2, class II, locus Mb1
15000	*H2-DMb2*	histocompatibility 2, class II, locus Mb2
15001	*H2-Oa*	histocompatibility 2, O region alpha locus
15002	*H2-Ob*	histocompatibility 2, O region beta locus
16161	*Il12rb1*	interleukin 12 receptor, beta 1
16184	*Il2ra*	interleukin 2 receptor, alpha chain
16185	*Il2rb*	interleukin 2 receptor, beta chain
16186	*Il2rg*	interleukin 2 receptor, gamma chain
16797	*Lat*	linker for activation of T cells
16818	*Lck*	lymphocyte protein tyrosine kinase
18037	*Nfkbie*	nuclear factor of kappa light polypeptide gene enhancer in B cells inhibitor, epsilon
18131	*Notch3*	notch 3
18761	*Prkcq*	protein kinase C, theta
19057	*Ppp3cc*	protein phosphatase 3, catalytic subunit, gamma isoform
19094	*Mapk11*	mitogen-activated protein kinase 11
20849	*Stat4*	signal transducer and activator of transcription 4
22637	*Zap70*	zeta-chain (TCR) associated protein kinase
381091	*H2-Eb2*	histocompatibility 2, class II antigen E beta2

## References

[1] Ford A.C., Sperber A.D., Corsetti M., Camilleri M. (2020). Irritable bowel syndrome. Lancet.

[2] Mearin F., Lacy B.E., Chang L., Chey W.D., Lembo A.J., Simren M., Spiller R. (2016). Bowel Disorders. Gastroenterology.

[3] Drossman D.A., Hasler W.L. (2016). Rome IV-Functional GI Disorders: Disorders of Gut-Brain Interaction. Gastroenterology.

[4] Zamani M., Alizadeh-Tabari S., Zamani V. (2019). Systematic review with meta-analysis: The prevalence of anxiety and depression in patients with irritable bowel syndrome. Aliment. Pharmacol. Ther..

[5] Sperber A.D., Bangdiwala S.I., Drossman D.A., Ghoshal U.C., Simren M., Tack J., Whitehead W.E., Dumitrascu D.L., Fang X., Fukudo S. (2021). Worldwide Prevalence and Burden of Functional Gastrointestinal Disorders, Results of Rome Foundation Global Study. Gastroenterology.

[6] Lovell R.M., Ford A.C. (2012). Global prevalence of and risk factors for irritable bowel syndrome: A meta-analysis. Clin. Gastroenterol. Hepatol..

[7] Carroll I.M., Ringel-Kulka T., Siddle J.P., Ringel Y. (2012). Alterations in composition and diversity of the intestinal microbiota in patients with diarrhea-predominant irritable bowel syndrome. Neurogastroenterol. Motil..

[8] Herndon C.C., Wang Y., Lu C. (2020). Targeting the gut microbiota for the treatment of irritable bowel syndrome. Kaohsiung J. Med. Sci..

[9] Jia Q., Zhang L., Zhang J., Pei F., Zhu S., Sun Q., Duan L. (2019). Fecal Microbiota of Diarrhea-Predominant Irritable Bowel Syndrome Patients Causes Hepatic Inflammation of Germ-Free Rats and Berberine Reverses It Partially. Biomed Res. Int..

[10] Chen M., Yuan L., Xie C., Wang X., Feng S., Xiao X., Zheng H. (2023). Probiotics for the management of irritable bowel syndrome: A systematic review and three-level meta-analysis. Int. J. Surg..

[11] Ducrotte P., Sawant P., Jayanthi V. (2012). Clinical trial: *Lactobacillus plantarum* 299v (DSM 9843) improves symptoms of irritable bowel syndrome. World J. Gastroenterol..

[12] Ait-Belgnaoui A., Payard I., Rolland C., Harkat C., Braniste V., Theodorou V., Tompkins T.A. (2018). *Bifidobacterium longum* and *Lactobacillus helveticus* Synergistically Suppress Stress-related Visceral Hypersensitivity Through Hypothalamic-Pituitary-Adrenal Axis Modulation. J. Neurogastroenterol. Motil..

[13] Liu Y., Xiao W., Yu L., Tian F., Wang G., Lu W., Narbad A., Chen W., Zhai Q. (2021). Evidence from comparative genomic analyses indicating that *Lactobacillus*-mediated irritable bowel syndrome alleviation is mediated by conjugated linoleic acid synthesis. Food Funct..

[14] Zhang H., Xia Y., Wang G., Xiong Z., Wei G., Liao Z., Qian Y., Cai Z., Ai L. (2024). *Lactobacillus plantarum* AR495 improves colonic transport hyperactivity in irritable bowel syndrome through tryptophan metabolism. Food Funct..

[15] Xia B., Lin T., Li Z., Wang J., Sun Y., Wang D., Ye J., Zhang Y., Kou R., Zhao B. (2024). *Lactiplantibacillus plantarum* Regulates Intestinal Physiology and Enteric Neurons in IBS through Microbial Tryptophan Metabolites. J. Agric. Food Chem..

[16] Liu Y., Yu X., Yu L., Tian F., Zhao J., Zhang H., Qian L., Wang Q., Xue Z., Zhai Q. (2021). *Lactobacillus plantarum* CCFM8610 Alleviates Irritable Bowel Syndrome and Prevents Gut Microbiota Dysbiosis: A Randomized, Double-Blind, Placebo-Controlled, Pilot Clinical Trial. Engineering.

[17] Aguilera-Lizarraga J., Hussein H., Boeckxstaens G.E. (2022). Immune activation in irritable bowel syndrome: What is the evidence?. Nat. Rev. Immunol..

[18] Luo R., Yao Y., Chen Z., Sun X. (2025). An examination of the LPS-TLR4 immune response through the analysis of molecular structures and protein-protein interactions. Cell Commun. Signal..

[19] Sun Y., Zhou J., Du H., Zhou Z., Han Y., Luo M., Guo X., Gu M., Yang H., Xiao H. (2024). The Anti-inflammatory Potential of a Strain of Probiotic *Bifidobacterium pseudocatenulatum* G7: In Vitro and In Vivo Evidence. J. Agric. Food. Chem..

[20] Al-Chaer E.D., Kawasaki M., Pasricha P.J. (2000). A new model of chronic visceral hypersensitivity in adult rats induced by colon irritation during postnatal development. Gastroenterology.

[21] Schmittgen T.D., Livak K.J. (2008). Analyzing real-time PCR data by the comparative C(T) method. Nat. Protoc..

[22] Staudacher H.M., Black C.J., Teasdale S.B., Mikocka-Walus A., Keefer L. (2023). Irritable bowel syndrome and mental health comorbidity-approach to multidisciplinary management. Nat. Rev. Gastroenterol. Hepatol..

[23] Mitselou A., Grammeniatis V., Varouktsi A., Papadatos S.S., Katsanos K., Galani V. (2020). Proinflammatory cytokines in irritable bowel syndrome: A comparison with inflammatory bowel disease. Intest. Res..

[24] Hanning N., Edwinson A.L., Ceuleers H., Peters S.A., De Man J.G., Hassett L.C., De Winter B.Y., Grover M. (2021). Intestinal barrier dysfunction in irritable bowel syndrome: A systematic review. Ther. Adv. Gastroenterol..

[25] Moloney R.D., O′Mahony S.M., Dinan T.G., Cryan J.F. (2015). Stress-induced visceral pain: Toward animal models of irritable-bowel syndrome and associated comorbidities. Front. Psychiatry.

[26] Barbara G., Wang B., Stanghellini V., de Giorgio R., Cremon C., Di Nardo G., Trevisani M., Campi B., Geppetti P., Tonini M. (2007). Mast cell-dependent excitation of visceral-nociceptive sensory neurons in irritable bowel syndrome. Gastroenterology.

[27] Sikander A., Rana S.V., Prasad K.K. (2009). Role of serotonin in gastrointestinal motility and irritable bowel syndrome. Clin. Chim. Acta.

[28] Zhou S., Gillilland M.R., Wu X., Leelasinjaroen P., Zhang G., Zhou H., Ye B., Lu Y., Owyang C. (2018). FODMAP diet modulates visceral nociception by lipopolysaccharide-mediated intestinal inflammation and barrier dysfunction. J. Clin. Investig..

[29] Karczewski J., Troost F.J., Konings I., Dekker J., Kleerebezem M., Brummer R.M., Wells J.M. (2010). Regulation of human epithelial tight junction proteins by *Lactobacillus plantarum* in vivo and protective effects on the epithelial barrier. Am. J. Physiol. –Gastroint. Liver Physiol..

[30] Anderson R.C., Cookson A.L., McNabb W.C., Park Z., McCann M.J., Kelly W.J., Roy N.C. (2010). *Lactobacillus plantarum* MB452 enhances the function of the intestinal barrier by increasing the expression levels of genes involved in tight junction formation. Bmc Microbiol..

[31] Rajilic-Stojanovic M., Jonkers D.M., Salonen A., Hanevik K., Raes J., Jalanka J., de Vos W.M., Manichanh C., Golic N., Enck P. (2015). Intestinal microbiota and diet in IBS: Causes, consequences, or epiphenomena?. Am. J. Gastroenterol..

[32] Akiho H., Ihara E., Motomura Y., Nakamura K. (2011). Cytokine-induced alterations of gastrointestinal motility in gastrointestinal disorders. World J. Gastrointest. Pathophysiol..

[33] Barbaro M.R., Cremon C., Marasco G., Savarino E., Guglielmetti S., Bonomini F., Palombo M., Fuschi D., Rotondo L., Mantegazza G. (2024). Molecular Mechanisms Underlying Loss of Vascular and Epithelial Integrity in Irritable Bowel Syndrome. Gastroenterology.

[34] Duboc H., Rainteau D., Rajca S., Humbert L., Farabos D., Maubert M., Grondin V., Jouet P., Bouhassira D., Seksik P. (2012). Increase in fecal primary bile acids and dysbiosis in patients with diarrhea-predominant irritable bowel syndrome. Neurogastroenterol. Motil..

[35] Wei W., Wang H., Zhang Y., Zhang Y., Niu B., Yao S. (2020). Altered metabolism of bile acids correlates with clinical parameters and the gut microbiota in patients with diarrhea-predominant irritable bowel syndrome. World J. Gastroenterol..

[36] Recharla N., Geesala R., Shi X. (2023). Gut Microbial Metabolite Butyrate and Its Therapeutic Role in Inflammatory Bowel Disease: A Literature Review. Nutrients.

[37] Osawa M., Handa O., Fukushima S., Matsumoto H., Umegaki E., Inoue R., Naito Y., Shiotani A. (2023). Reduced abundance of butyric acid-producing bacteria in the ileal mucosa-associated microbiota of ulcerative colitis patients. J. Clin. Biochem. Nutr..

[38] Gomez-Arango L.F., Barrett H.L., McIntyre H.D., Callaway L.K., Morrison M., Dekker Nitert M. (2016). Increased Systolic and Diastolic Blood Pressure Is Associated With Altered Gut Microbiota Composition and Butyrate Production in Early Pregnancy. Hypertension.

